# Prognostic value of low microRNA-34a expression in human gastrointestinal cancer: a systematic review and meta-analysis

**DOI:** 10.1186/s12885-020-07751-y

**Published:** 2021-01-14

**Authors:** Yan-Ling Chen, Xiao-Lin Liu, Ling Li

**Affiliations:** Department of Gastroenterology, The First Affiliated Hospital of Soochow University, 899 Ping Hai Road, Suzhou, 215006 Jiangsu China

**Keywords:** microRNA-34a, Gastrointestinal cancer, Prognosis, Meta-analysis

## Abstract

**Background:**

Mounting evidence shows that microRNA-34a (miR-34a) is involved in cancer prognosis. Therefore, we summarize the predictive role of miR-34a for survival in patients with gastrointestinal cancers (GICs).

**Methods:**

All eligible studies were found by searching PubMed, Web of Science and EMBASE, and survival results were extracted. Then, the hazard ratio (HR) with the corresponding 95% confidence interval (CI) was calculated to evaluate the prognostic role of miR-34a in GICs. The association between miR-34a expression and clinicopathological characteristics was estimated by odds ratios (ORs) and 95% CIs.

**Results:**

A total of 20 studies were included in this meta-analysis. For overall survival (OS), lower miR-34a expression could probably predict poorer outcome in GICs, with a pooled HR of 1.86 (95% CI: 1.52–2.28, *P* < 0.01). For disease-free survival (DFS), progression-free survival (PFS), and recurrence-free survival (RFS), lower miR-34a expression was related to worse DFS/PFS/RFS with a pooled HR of 1.86 (95% CI: 1.31–2.63, *P*  <  0.01). A significant relation of differentiation/TNM stage/lymphatic metastasis and the expression level of miR-34a was identified.

**Conclusion:**

This meta-analysis revealed that lower miR-34a expression is significantly connected with worse OS and DFS/PFS/RFS in GIC patients. In addition, the miR-34a expression level is relatively lower in patients with lymph node metastasis than in patients without lymph node metastasis, and decreased miR-34a expression levels are linked to poor tumour differentiation and late TNM stage. MiR-34a may become a new factor for the prognosis prediction and progression of GICs.

**Supplementary Information:**

The online version contains supplementary material available at 10.1186/s12885-020-07751-y.

## Background

Gastrointestinal cancers (GICs) account for the majority of cancer-related deaths worldwide, especially in developing countries [1]. Previous studies have shown that stomach, oesophageal, liver, and colorectal cancers are commonly identified as the leading causes of cancer deaths [2]. Currently, common treatments for GICs include surgery, neoadjuvant chemoradiotherapy, adjuvant chemoradiotherapy and immunotherapy; however, the therapeutic effects are limited in patients at advanced stages. Therefore, there is an urgent need for early detection of GICs and recognition of high-risk patients with poor prognosis.

MicroRNAs (miRNAs) are small-molecule RNAs with a length of 19 to 25 nucleotides that regulate the posttranscriptional silencing of target genes by combining with the 3′-untranslated region (3′-UTR) of target messenger RNA [3]. MiRNAs participate in various biological processes, including cell multiplication, differentiation, apoptosis and cell cycle regulation [4]. Studies have reported that miRNAs are abnormally expressed in tumours and have strong diagnostic and prognostic values [5].

MicroRNA-34a (miR-34a), a member of the miR-34 family, has been verified to be abnormally expressed in various tumours, including oesophageal cancer (EC) [6], gastric cancer (GC) [7], colorectal cancer (CRC) [8], hepatocellular carcinoma (HCC) [9], pancreatic cancer (PC) [10], gallbladder cancer (GBC) [11], and other cancers [12]. Based on recent studies, miR-34a has been considered closely related to gastrointestinal cancer multiplication [13], invasion [14] and metastasis [15], which points to the important biological roles of miR-34a in cellular signalling pathways, such as the MAPK/Ras pathway [16], Wnt/β-Catenin pathway [17], PI3K/Akt pathway [18], SIRT1/p53 pathway [19], and FoxM1/c-Myc pathway [20]. However, the prognostic accuracy of miR-34a in GICs was inconsistent among these studies. Hu et al. [21], Hui et al. [22], and Yang et al. [23] reported that a low expression level of miR-34a predicted a worse survival rate in GIC patients. In contrast, Osawa et al. [24], Zhang et al. [25] and Mojin Wang et al. [26] found that GIC patients benefited from downregulated miR-34a expression. To assess the prognostic value of miR-34a in GICs systematically and to discuss the association between miR-34a expression and clinicopathological characteristics, we performed a meta-analysis on the basis of all published relevant studies.

## Methods

### Literature search

We searched the PubMed, Web of Science and Embase databases to identify relevant studies before January 1, 2020. The following keywords were used: ‘microRNA-34a’, ‘miR-34a’, ‘cancer’, ‘neoplasm’, ‘oesophageal’, ‘stomach’, ‘colorectal’, ‘colon’, ‘pancreatic’, ‘hepatocellular’, ‘liver’, ‘gallbladder’, ‘prognosis’, ‘survival’, ‘hazard ratio’, and ‘gastrointestinal’. These keywords were combined with ‘AND’ or ‘OR’. The results were limited to papers published in English.

### Selection criteria

Studies were included based on the following conditions: (1) the diagnosis of GICs was confirmed by histopathology; (2) the expression of miR-34a in tissue or blood was measured and divided into high and low levels; and (3) the survival outcome was reported directly or survival data were provided from Kaplan-Meier survival curves. The exclusion criteria were as follows: (1) reviews, laboratory studies or letters; and (2) the lack of or inability to calculate key information about survival outcomes, such as the HR or 95% CI.

### Data extraction and quality assessment

Two investigators (Yan-Ling Chen and Xiao-Lin Liu) independently extracted the data from all eligible references, including first author, publication time, country, tumour type, sample type, test method, TNM stage, follow-up time and cut-off value, HRs of miR-34a for OS and/or DFS, PFS, RFS, and 95% CIs. In addition, data on clinical characteristics were collected from the studies that reported such information. All eligible studies were retrospective. The Newcastle-Ottawa Scale (NOS) was used to assess the quality of each study. The range of scores is 0 to 9, and a score greater than 6 was considered high quality [27]. Any disagreement was finally resolved by discussion.

### Statistical analysis

We used RevMan 5.3 (Cochrane Collaboration, Oxford, UK) and STATA 12.0 (StataCorp LP, College Station, TX, USA) to conduct the statistical analysis. The pooled HRs and corresponding 95% CIs were used to evaluate the prognostic value of low miR-34a expression in GICs. The heterogeneity among studies was calculated by Cochran’s Q test and Higgins’s I^2^ statistic. If *P*  >  0.05 or I^2^ ≤ 50%, we considered no significant heterogeneity to exist, and a fixed-effect model was used; if *P* ≤ 0.05 or I^2^  >  50%, a random-effect model was used. Some studies did not provide the HRs and 95% CIs directly, and we obtained the key points and the relevant data from Kaplan-Meier survival curves by utilising Engauge Digitizer 4.1 software and then calculated the HR and corresponding 95% CI following Tierney’s method [28]. Publication bias was assessed by funnel plots and Egger’s test. In addition, we performed a sensitivity analysis by removing studies one by one to assess the influence of a single study. The association between miR-34a expression and clinicopathological characteristics was evaluated by the pooled OR and 95% CI.

## Results

### Literature search

**Fig. 1:**
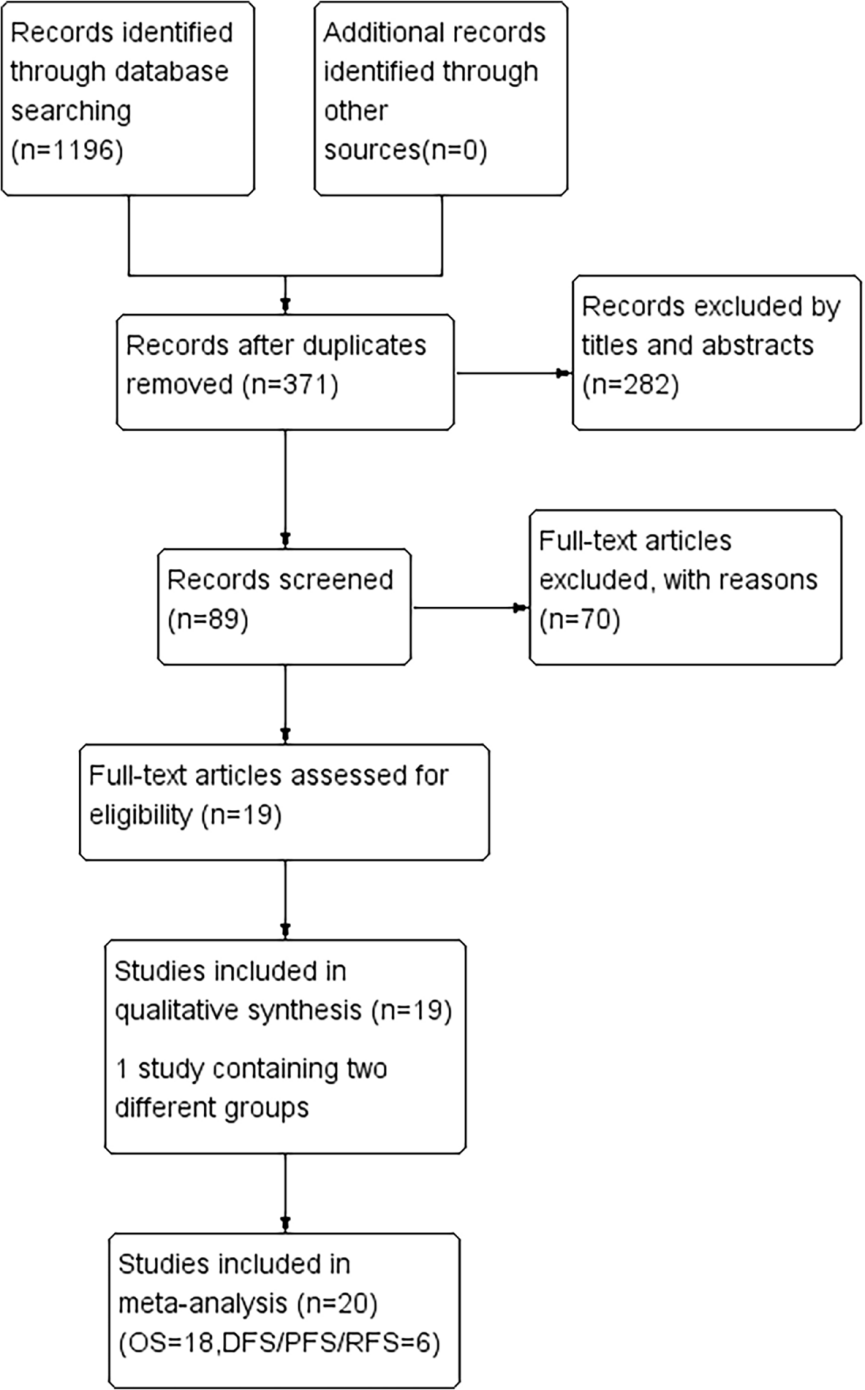
Flow diagram of the study selection process

A total of 1196 records were obtained in the beginning. A total of 825 studies were excluded because of duplication, and 282 records were excluded after screening the titles and abstracts. According to the selection criteria, 19 studies were finally identified as eligible, including 2 EC, 5 GC, 4 HCC, 4 PC, 3 CRC, and 1 GBC. Since one of the studies contained two different groups, 20 independent experiments were included for quantitative analysis. The flow diagram of the study selection is shown in Fig. 1.

### Characteristics of the eligible studies

The main features of eligible studies are summarized in Table 1, and the summary of HRs and their 95% CIs are shown in Table 2. The eligible articles were published between 2011 and 2019, including 1691 participants with OS data and 676 participants with DFS/PFS/RFS data from China, America, Japan, Scotland, and Slovakia. The types of GICs included EC, GC, CRC, HCC, PC, and GBC. Quantitative real-time PCR (qRT-PCR) was extensively used in all studies to assess the expression of miR-34a. Tumour tissues were the most commonly used sample, except for Long L-M’s study [10] in which plasma samples were used. Among the included studies, 8 studies reported HRs and the corresponding 95% CIs directly, and the HRs and 95% CIs of the remaining 12 studies were calculated by Kaplan-Meier survival curves.

**Table 1:** The main characteristics of included 20 studies

Study	Year	Country	Type	Design	Sample	Num.	Stage	Cut-off	Follow-up	Test method	Outcome
Hu et al. [21]	2011	America	EC	R	Tissue	99	I-IV	Median	> 250	qRT-PCR	OS/DFS
Lin et al. [29]	2015	China	EC	R	Tissue	111	I-IV	Median	NR	qRT-PCR	OS
Osawa et al. [24]	2011	Japan	GC	R	Tissue	37	II-III	70%	60	qRT-PCR	OS
Hui et al. [22]	2015	China	GC	R	Tissue	76	I-III	Mean	> 60	qRT-PCR	OS
Wei et al. [30]	2015	China	GC	R	Tissue	157	I-IV	NR	> 100	qRT-PCR	OS
Zhang et al. [31]	2015	China	GC	R	Tissue	137	I-IV	2.44	68	qRT-PCR	OS
Yang et al. [23]	2015	China	GC	R	Tissue	50	I-IV	Median	60	qRT-PCR	OS
Li et al. [32]	2015	China	HCC	R	Tissue	114	I-IV	ROC	90	qRT-PCR	OS/PFS
Yang et al. [33]	2013	China	HCC	R	Tissue	30	NR	Mean	60	qRT-PCR	OS
Cui et al. [34]	2015	China	HCC	R	Tissue	120	NR	Median	60	qRT-PCR	OS/RFS
Xu et al. [20]	2015	China	HCC	R	Tissue	75	I-IV	Median	60	qRT-PCR	OS/RFS
Ohuchida et al. [35]	2011	Japan	PC	R	Tissue	90	NR	NR	>100	qRT-PCR	OS
Jamieson et al. [36]	2012	Scotland	PC	R	Tissue	72	NR	Median	48	qRT-PCR	OS
Long et al. [10]	2016	China	PC	R	plasma	159	I-IV	Mean	24	qRT-PCR	OS
Sun et al. [37]	2018	China	PC	R	Tissue	139	I-IV	Mean	60	qRT-PCR	OS
Zhang et al. [25]	2017	China	CRC	R	Tissue	84	I-IV	2	36	qRT-PCR	OS
Hasakova et al. [38]	2019	Slovakia	CRC	R	Tissue	64	I-IV	Median	100	qRT-PCR	OS
Gao et al. [39]	2014	China	CRC	R	Tissue	205	II-III	0.307	>80	qRT-PCR	DFS
Gao et al. [39]	2014	China	CRC	R	Tissue	63	II-III	0.307	>80	qRT-PCR	DFS
Jin et al. [11]	2013	China	GBC	R	Tissue	77	NR	Mean	24	qRT-PCR	OS

*Abbreviations*: *CRC* colorectal cancer; *DFS* disease-free survival, *EC* esophageal cancer; *GBC* gallbladder cancer; *GC* gastric cancer; *HCC* hepatocellular carcinoma, *NR* no report, *OS* overall survival, *PC* pancreatic cancer, *PFS* progressive-free survival, *qRT-PCR* quantitative real-time PCR, *R* retrospective, *RFS* recurrence-free survival

**Table 2:** Summary of HRs and their 95% CI

Study	Year	Country	Tumor type	Outcome	HR	95% CI	NOS
Hu et al. [21]	2011	America	EC	OS DFS	1.41 1.39	0.81–2.44 0.82–2.35	8
Lin et al. [29]	2015	China	EC	OS	3.182	1.273–10.184	6
Osawa et al. [24]	2011	Japan	GC	OS	0.2	0.06–0.68	6
Hui et al. [22]	2015	China	GC	OS	2.327	1.099–4.927	7
Wei et al. [30]	2015	China	GC	OS	2.31	0.13–40.12	8
Zhang et al. [31]	2015	China	GC	OS	1.33	1.14–1.61	8
Yang et al. [23]	2015	China	GC	OS	3.05	0.6–15.50	8
Li et al. [32]	2015	China	HCC	OS PFS	1.81 1.22	1.03–3.18 0.92–1.62	6
Yang et al. [33]	2013	China	HCC	OS	3.54	1.67–7.52	7
Cui et al. [34]	2015	China	HCC	OS RFS	1.44 1.49	1.13–1.72 1.15–1.79	8
Xu et al. [20]	2015	China	HCC	OS RFS	1.96 1.96	1.04–3.57 1.10–3.45	8
Ohuchida et al. [35]	2011	Japan	PC	OS	2.92	1.303–6.295	8
Jamieson et al. [36]	2012	Scotland	PC	OS	6.67	2.684–16.573	8
Long et al. [10]	2016	China	PC	OS	1.88	1.35–2.64	8
Sun et al. [37]	2018	China	PC	OS	2.24	1.38–3.36	7
Zhang et al. [25]	2017	China	CRC	OS	1.76	1.01–3.05	6
Hasakova et al. [38]	2019	Slovakia	CRC	OS	1.34	0.65–2.75	8
Gao et al. [39]	2014	China	CRC	DFS DFS	3.819 2.973	2.438–5.983 1.339–6.602	8
Jin et al. [11]	2013	China	GBC	OS	2.37	1.11–5.06	8

*Abbreviations*: *95%CI* 95% confidence interval, *CRC* colorectal cancer, *DFS* disease-free survival, *EC* esophageal cancer, *GBC* gallbladder cancer, *GC* gastric cancer, *HCC* hepatocellular carcinoma, *HR* hazard ratio, *OS* overall survival, *PC* pancreatic cancer, *PFS* progressive-free survival, *RFS* recurrence-free survival

### Overall survival is associated with miR-34a expression

**Fig. 2:**
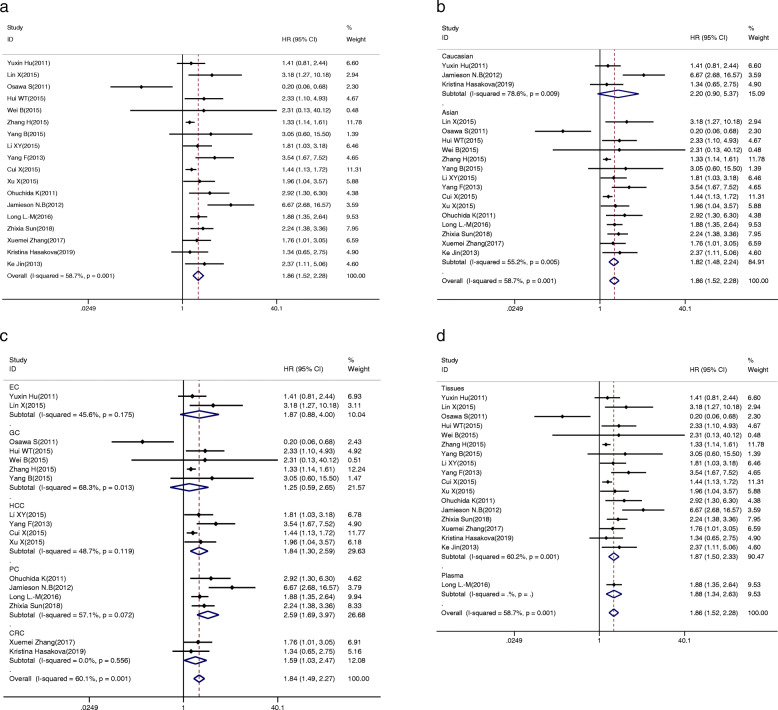
The association between miR-34a expression levels and (**a**) overall survival; subgroup analyses of (**b**) ethnicity (Asian and Caucasian), **c** cancer type (EC, GC, HCC, PC, CRC), and (**d**) specimen (plasma and tissues)

We analysed the association between low expression of miR-34a and OS at first, and remarkable heterogeneity between studies was found (I^2^ = 58.7%, *P* = 0.001, Table 3). Therefore, the random-effect model was used to compute the pooled HR and corresponding 95% CI. The result showed that a lower expression level of miR-34a significantly predicted worse OS, with a pooled HR of 1.86 (95% CI: 1.52–2.28; Fig. 2a).

**Table 3:** Association between miR-34a expression levels and overall survivals

	No.of studies	No.of patients	Pooled HR (95%CI)		Meta regression *p*-value	Heterogeneity	
			Fixed	Random		I^2^	*p*-value
Overall	18	1691	1.600 (1.44–1.77)	1.86 (1.52–2.28)		58.7%	0.001
Ethnicity					0.806		
Asian	15	1456	1.58 (1.42–1.76)	1.82 (1.48–2.24)		55.2%	0.005
Caucasian	3	235	1.86 (1.25–2.76)	2.20 (0.90–5.37)		78.6%	0.009
Sample Size					0.979		
≥ 100	7	937	1.51 (1.34–1.69)	1.61 (1.35–1.92)		36.1%	0.153
<100	11	754	1.98 (1.59–2.48)	2.00 (1.37–2.93)		63.2%	0.002
NOS Scores					0.978		
≥ 8	11	1100	1.53 (1.36–1.71)	1.75 (1.42–2.16)		49.5%	0.031
< 8	7	591	2.00 (1.56–2.55)	1.87 (1.20–2.93)		65.8%	0.008
Specimen					0.933		
tissue	17	1532	1.57 (1.41–1.75)	1.87 (1.50–2.33)		60.2%	0.001
plasma	1	159	1.88 (1.34–2.63)	1.88 (1.34–2.63)		–	–
Cancer Types					0.494		
EC	2	210	1.69 (1.04–2.74)	1.87 (0.88–4.00)		45.6%	0.175
GC	5	457	1.33 (1.13–1.57)	1.25 (0.59–2.65)		68.3%	0.013
HCC	4	339	1.60 (1.33–1.92)	1.84 (1.30–2.59)		48.7%	0.119
PC	4	460	2.27 (1.77–2.89)	2.59 (1.69–3.97)		57.1%	0.072
CRC	2	148	1.59 (1.03–2.47)	1.59 (1.03–2.47)		0.0%	0.556
GBC	1	77	2.37 (1.11–5.06)	2.37 (1.11–5.06)		–	–

*Abbreviations*: *95%CI* 95% confidence interval, *CRC* colorectal cancer, *EC* esophageal cancer, *GBC* gallbladder cancer, *GC* gastric cancer, *HCC* hepatocellular carcinoma, *HR* hazard ratio, *NOS* Newcastle-Ottawa Scale, *PC* pancreatic cancer

To explicate the heterogeneity in OS, subgroup analysis was conducted by ethnicity (Asian and Caucasian), sample capacity (≥100 and < 100), NOS scores (≥8 and < 8), specimen (plasma and tissue) and tumour types (EC, GC, CRC, HCC and PC). As a result, homogeneity was achieved in the CRC group (I^2^ = 0.00%, *P* = 0.556; Table 3) and the correlation was obvious (HR  =  1.59, 95% CI:1.03–2.47, Fig. 2c). Additionally, there were significant correlations between the expression level of miR-34a and OS in Asian populations (HR  =  1.82, 95% CI: 1.48–2.24, Fig. 2b); a sample capacity greater than or equal to 100 (HR = 1.61, 95% CI: 1.35–1.92, Supplementary Fig. 1A) or less than 100 (HR = 2.00, 95% CI: 1.37–2.93, Supplementary Fig. 1A); NOS scores equal to or greater than 8 (HR = 1.75, 95% CI: 1.42–2.16, Supplementary Fig. 1B) or less than 8 (HR = 1.87, 95% CI: 1.20–2.93, Supplementary Fig. 1B); specimens removed the plasma (HR = 1.87, 95% CI: 1.50–2.33, Fig. 2d), HCC (HR  =  1.84, 95% CI: 1.30–2.59, Fig. 2c), and PC (HR  =  2.59, 95% CI:1.69–3.97, Fig. 2c) by the random-effect model. As shown in Table 3, the significance disappeared in Caucasian and EC groups when the fixed-effect model was transformed into the random-effect model. Moreover, the heterogeneities were still evident among subgroups, except for the CRC group. Ultimately, to analyse heterogeneity, meta regression was performed, but it was unable to explain the variation in HRs (*p* = 0.806 for ethnicity, *p* = 0.979 for sample capacity, *p* = 0.978 for NOS scores, *p* = 0.933 for specimen, and *p* = 0.494 for cancer types, Table 3). Moreover, the sensitivity analysis was performed to assess the contribution of each study, and no study seemed to make a difference to the pooled results (Supplementary Fig. 2A). In addition, publication bias was evaluated by funnel plots and Egger’s test. As shown in Supplementary Fig. 2B, the funnel plots showed no obvious asymmetry, and Egger’s test revealed that no significant publication bias existed (*P* = 0.058).

### Tumour progression is associated with miR-34a expression

**Fig. 3:**
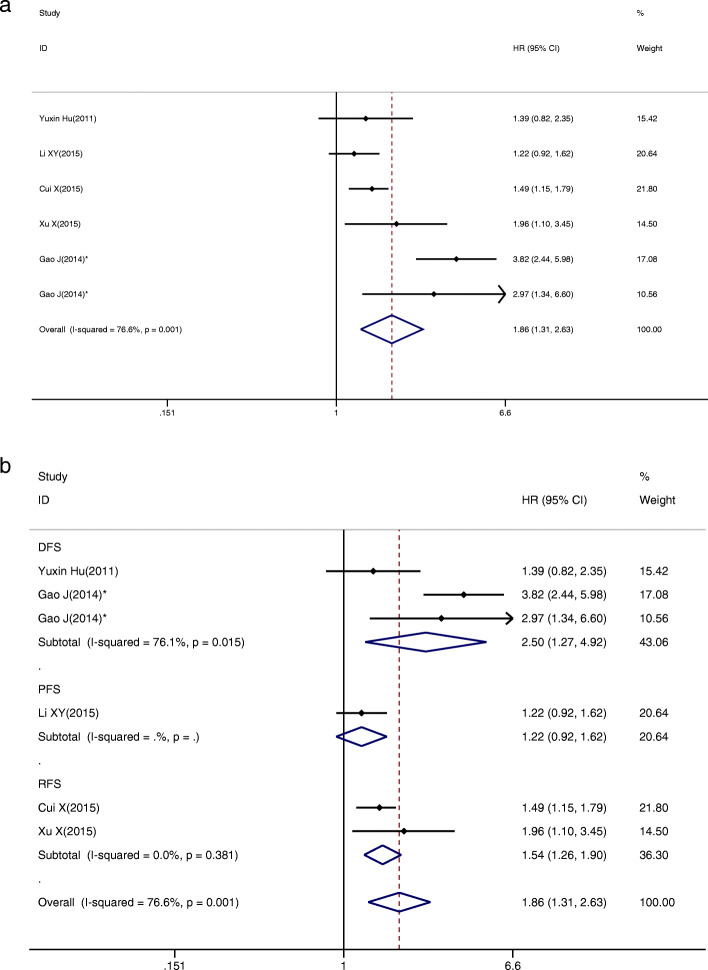
The association between miR-34a expression levels and (**a**) DFS/PFS/RFS; **b** subgroup analyses of DFS/PFS/RFS. Gao J*, study containing two different groups

To evaluate the association between miR-34a expression and DFS/PFS/RFS, 6 studies were included in this analysis, and the data revealed that low miR-34a expression predicted a worse outcome with a combined HR of 1.86 (95% CI: 1.31–2.63) via a random-effect model (*P* = 0.001, I^2^ = 76.6%; Fig. 3a). To explain the heterogeneity, we performed subgroup analysis by DFS, PFS and RFS, showing a significant correlation with the expression of miR-34a (HR  =  2.50, 95% CI: 1.27–4.92 for DFS; HR  =  1.54, 95% CI: 1.26–1.90 for RFS; Fig. 3b). Moreover, homogeneity was achieved in the RFS group. Then, the sensitivity analysis was performed by removing studies one by one to assess the influence of a single study. As shown in Supplementary Fig. 2C, the stability of the entire study was not influenced by individual studies. Finally, funnel plots and Egger’s test were implemented to evaluate publication bias. The funnel plot was roughly symmetric (Supplementary Fig. 2D), and the *P* value of Egger’s test was 0.909. Therefore, no evidence for significant publication bias existed.

### Correlation between miR-34a levels and clinicopathological features in GICs

**Fig. 4:**
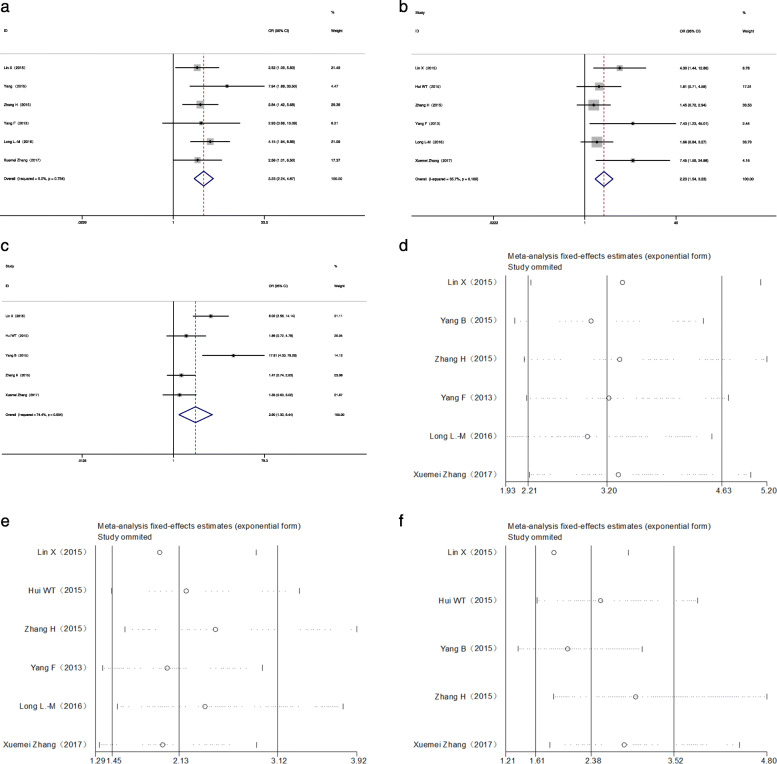
The association between miR-34a expression levels and (**a**) lymphatic metastasis, (**b**) tumour differentiation degree, and (**c**) TNM stage; sensitivity analyses for ORs of clinicopathological characteristics, such as (**d**) lymphatic metastasis, (**e**) tumour differentiation degree, and (**f**) TNM stage

For obtaining relevant statistics to evaluate the relation between miR-34a expression levels and different clinicopathological characteristics, seven studies containing 647 patients with GICs were screened out. As shown in Table 4, we observed a significant association between the expression level of miR-34a and lymphatic metastasis (OR = 3.231, 95% CI: 2.237–4.666; Fig. 4a) and differentiation degree (OR = 2.228, 95% CI: 1.538–3.228; Fig. 4b) via the fixed-effect model, as well as TNM stage (OR = 2.896, 95% CI: 1.302–6.442; Fig. 4c) via the random-effect model. There was no significant correlation identified between miR-34a level and tumour size (OR = 0.736, CI: 0.460–1.177). In addition, the expression level of miR-34a was unaffected by gender (OR = 0.776, 95% CI: 0.566–1.065). The heterogeneity disappeared in the gender group (I^2^ = 0.00%, *P* = 0.888), lymphatic metastasis group (I^2^ = 0.00%, *P* = 0.754), medium level of the tumour size group (I^2^ = 20.5%, *P* = 0.284), and differentiation degree group (I^2^ = 35.7%, *P* = 0.169), but it was obvious in the TNM stage group (I^2^ = 74.4%, *P* = 0.004). Sensitivity analysis was applied to assess the stability, including lymphatic metastasis (Fig. 4d), differentiation degree (Fig. 4e) and TNM stage (Fig. 4f), suggesting no study had significant impact on the results.

**Table 4:** Overall analysis of miR-34a expression association with clinicopathologic characteristics

Clinicopathological characteristics	Num. of studies	Num. of patients	Pooled OR (95%CI)		Heterogeneity	
			Fixed	Random	I^2^	*p*-value
Gender (male vs. female)	7	647	0.776 (0.566–1.065)	0.777 (0.565–1.067)	0.0%	0.888
Tumor Size (≤5 vs > 5 cm)	3	326	0.736 (0.460–1.177)	0.284 (0.433–1.288)	20.5%	0.284
Lymphatic Metastasis (YESvs.NO)	6	571	3.231 (2.237–4.666)	3.200 (2.210–4.635)	0.0%	0.754
TNM stage (III + IV vs. I + II)	5	458	2.468 (1.698–3.588)	2.896 (1.302–6.442)	74.4%	0.004
Differentiation (poor vs. others)	6	597	2.228 (1.538–3.228)	2.373 (1.430–3.938)	35.7%	0.169

*Abbreviations*: *95%CI* 95% confidence interval, Fixed, fixed effects model, *OR* Odds ratio, Random, random pooling model

## Discussion

In the last few decades, miRNAs have attracted increasing interest among investigators as potential biomarkers for cancer diagnosis and prognosis. Many clinical trials have demonstrated that miRNAs play a pivotal role in tumour development by regulating the expression of target genes and tumour suppressors or directly performing their functions as oncogenes or tumour suppressors [40, 41]. MiR-34a expression is transcriptionally controlled by p53, a vital tumour suppressor pathway, which is disrupted in cancer frequently. It has been reported that miR-34a influenced tumour biological activities by targeting several genes or signal pathways, such as CCND1 in EC [42], PDGFR in GC [18], HMGB1 in CRC [43], and XIST in PC [37]. Recently, a systematic review has summarized numerous studies that reported the diagnostic and prognostic value of miR-34a in GICs [44]. However, among these studies, two opposing views were presented on whether patients could benefit from the high expression of miR-34a. Hao Wu et al. [7], Milad Asadi et al. [45] and Yan Zhou et al. [46] showed that the downregulation of miR-34a was linked to a poor prognosis in GIC patients, while Hiyoshi et al. [8], and Mojin Wang [26] reported that patients benefited from downregulated miR-34a. The prognostic value of miR-34a in GICs has been illustrated in many studies, but the particular prognostic role of miR-34a in GICs remains unclear. As far as we know, this is the most comprehensive meta-analysis exploring the clinical value of miR-34a in patients with GICs.

This meta-analysis discussed 20 papers and contained 2367 patients in total. Among these studies, 18 studies including 1691 patients provided the relevant OS statistics. By the random-effect model, the results showed that the decreased miR-34a expression was associated with poorer outcomes in patients with GICs. To explain the potential sources of heterogeneity, subgroup analyses were performed. As a result, homogeneity was reached in the CRC group, and the OS of the CRC group was found to be greatly associated with miR-34a expression levels. Though the expression level of miR-34a in CRC patients remains controversial, there are several potential mechanisms that suggest how low expression of miR-34a could induce an unfavourable outcome of CRC. MiR-34a expression is governed by p53 and could inhibit recurrence of CRC by inhibiting cell growth, migration and invasion and inducing cell apoptosis and cell cycle arrest in a p53-dependent manner [39]. Moreover, it has been reported that miR-34a served a key role in suppressing CRC metastasis by targeting and regulating Notch signalling [25] and the FMNL2 and E2F pathways [47, 48]. In addition, Jiang et al. showed that miR-34a suppressed tumour formation caused by loss of Apc and controlled intestinal stem cell and secretory cell homeostasis by downregulation of multiple target mRNAs, such as Pdgfra, Pdgfrb, and Axl [49].

As shown in Table 3, the associations between miR-34a expression levels and OS were also significant in other subgroups. In the included studies, mir-34a showed a low expression level in both tumour tissue and blood, except for Osawa’s study [24], in which tissue samples were used. Subgroup analysis showed that the miR-34a level from tissue (HR  =  1.87, 95% CI: 1.50–2.33) and plasma (HR  =  1.88, 95% CI: 1.34–2.63) were of equal importance in prognostic value. Since there was only one study included based on plasma samples, the conclusion remained unclear until now and required further verification. In addition, subgroup analysis of tumour types showed a closer relationship between a low miR-34a level and poor OS in patients with PC (HR  =  2.59, 95% CI:1.69–3.97). Empirically, HR > 2 is considered strongly predictive [50]. As for the possible mechanism, Long et al. reported that miR-34a significantly inhibited the tumour growth of PC tumours by suppressing Notch1, Notch2 and Notch4 expression [10]. Tang et al. found that the EMT program activator Snail1 and the proliferation regulator Notch1 were both targets of miR-34a [51]. Overexpression of miR-34a suppressed the expression of Snail1, which in turn upregulated E-cadherin. Moreover, the HDAC inhibitor Vorinostat (SAHA) inhibited the expression of EMT inducers Zeb-1, Snail, and Slug by upregulating the expression of miR-34a, thereby attenuating the migration and invasion of PC cells [52]. Since the heterogeneities within the subgroups were still significant, meta regression was performed to illustrate the influence of different factors, including ethnicity, sample capacity, specimen, NOS scores and tumour classification, but there was no factor that significantly affected the variation in HR. The analysis of tumour progression and miR-34a expression revealed that low miR-34a expression seemed to predict a worse outcome, especially in DFS (HR  =  2.50, 95% CI: 1.27–4.92). According to our research, we could infer that the decreased expression level of miR-34a was closely related to worse prognosis in patients with GICs. However, for the EC and GC groups, the results were still not stable and required more comprehensive studies to further research the miR-34a prognostic value in GICs.

To evaluate the association between miR-34a and the clinical characteristics, seven articles including 647 patients were included. Significant relations were observed between miR-34a expression levels and differentiation/TNM stage/lymphatic metastasis by a fixed- or random-effect model. Sensitivity analysis indicated that no study had a significant impact on the results. Based on the findings above, we could suggest that patients with decreased miR-34a expression are more likely to develop lymphatic metastasis, and decreased miR-34a expression level is linked to poor tumour differentiation and late TNM stage.

Though this meta-analysis revealed that miR-34a was a promising biomarker of GICs, several potential limitations of this study should be considered. First, the number of included studies was limited; the current sample size was too small to explain the real relationship between miR-34a expression level and prognosis of GICs. Subgroup analyses were also affected by the relative lack of studies; for example, there was only one article related to PFS. The significance of this study lies in larger sample size experiments for further identification of the correlation between miR-34a and prognosis of GICs. Second, patients were all Asian and Caucasian, and the lack of data from other regions might have resulted in ethnic bias. Third, the cut-off values among studies were different, and we did not have absolute criteria to assess whether the expression of miR-34a was low or not, thus impacting the statistical power of the analysis. Finally, several HRs and 95% CIs were calculated according to the data extracted from survival curves, so it is difficult to exclude the influence of confounding bias.

## Conclusion

In conclusion, our study demonstrates that lower miR-34a expression is significantly associated with poorer OS and DFS/PFS/RFS and may be a novel prognostic biomarker in GICs. Moreover, the miR-34a expression level is relatively lower in patients with lymph node metastasis, and a decreased expression level of miR-34a is related to poor tumour differentiation and late TNM stage. Further multicentre prospective clinical studies are needed to validate the association between miR-34a and the prognosis of GICs.

## Supplementary Information

**Additional file 1: Supplementary Figure 1.** The association between miR-34a expression levels and (A) sample size (≥100 and < 100) and (B) NOS scores (≥8 and < 8).

**Additional file 2: Supplementary Figure 2.** Sensitivity analysis for the HR of (A) OS; (C) DFS/PFS/RFS; publication bias evaluation for (B) OS; (D) DFS/PFS/RFS.

## Data Availability

The authors declare that all data used or analysed during the current study are available on reasonable request.

## Abbreviations

95% CI: 95% confidence interval
CRC: Colorectal cancer
DFS: Disease-free survival
EC: Oesophageal cancer
GBC: Gallbladder cancer
GC: Gastric cancer
HCC: Hepatocellular carcinoma
HR: Hazard ratio
NOS: Newcastle-Ottawa Scale
NR: No report
OR: Odds ratio
OS: Overall survival
PC: Pancreatic cancer
PFS: Progression-free survival
qRT-PCR: Quantitative real-time PCR
R: Retrospective
RFS: Recurrence-free survival
