# Supplementary figures and images for: Prognostic value of low microRNA-34a expression in human gastrointestinal cancer: a systematic review and meta-analysis
Source: BMC Cancer. 2021 Jan 14;21:63. doi: 10.1186/s12885-020-07751-y (PMC7807881; doi:10.1186/s12885-020-07751-y)

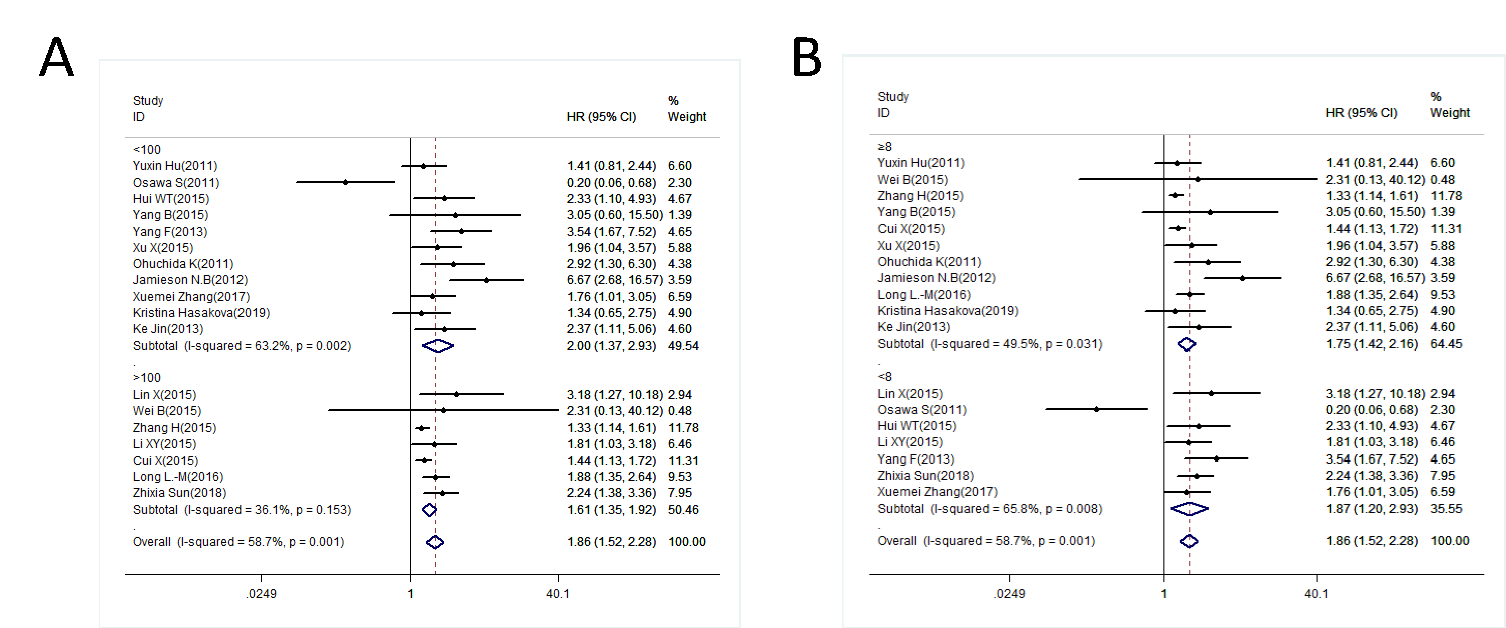

Supplement: Supplementary file 1 — Additional file 1: Supplementary Figure 1. The association between miR-34a expression levels and (A) sample size (≥100 and < 100) and (B) NOS scores (≥8 and < 8). [file 12885_2020_7751_MOESM1_ESM.tiff]

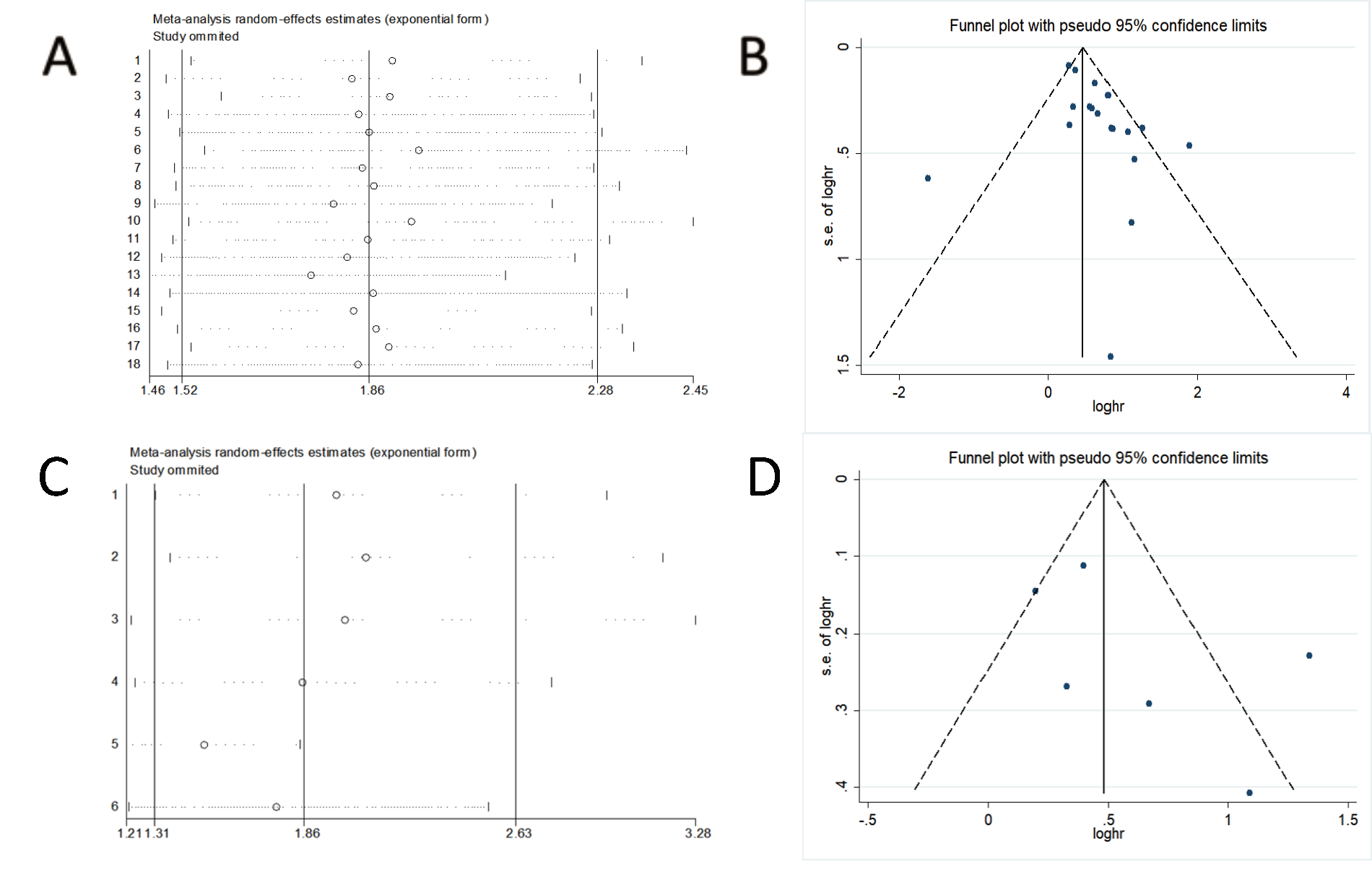

Supplement: Supplementary file 2 — Additional file 2: Supplementary Figure 2. Sensitivity analysis for the HR of (A) OS; (C) DFS/PFS/RFS; publication bias evaluation for (B) OS; (D) DFS/PFS/RFS. [file 12885_2020_7751_MOESM2_ESM.tiff]
